# Drug Repurposing in Veterinary Oncology: Myth or Reality?

**DOI:** 10.3390/vetsci12111067

**Published:** 2025-11-06

**Authors:** Stefano Ciccarelli, Chiara Perrone, Maria Alfonsa Cavalera, Antonio Giuliano

**Affiliations:** 1 Department of Veterinary Medicine, University of Bari “Aldo Moro”, 70010 Valenzano, Italy; stefano.ciccarelli@uniba.it (S.C.); mariaalfonsa.cavalera@uniba.it (M.A.C.); 2 Harvest Veterinary Oncology Center, Kwai Fung, Kwai Chung, Kowloon, Hong Kong 999077, China; dr.giuliano@hvoc.com.hk

**Keywords:** canine, comparative oncology, off-label drug use, translational research

## Abstract

**Simple Summary:**

Cancer is a leading cause of disease and death in companion animals, yet developing new anticancer drugs is expensive, time-consuming and often inaccessible for pet owners. Drug repurposing, which involves exploring new uses for existing compounds, offers a pragmatic and sustainable alternative. Relying on drugs with well-established safety profiles can provide more affordable therapies while accelerating their clinical availability. This review examines a selection of repurposed agents with potential applications in veterinary oncology including anti-inflammatory drugs (such as piroxicam), metabolic regulators (such as metformin), antiparasitic compounds (such as fenbendazole), cardiovascular medicines (such as propranolol and statins) and immunomodulators (such as thalidomide). Evidence from laboratory studies, spontaneous tumor models and preliminary clinical research suggests that these drugs may slow cancer progression, enhance the effects of chemotherapy or stimulate the immune response. Overall, drug repurposing holds promise for expanding cancer treatment options in dogs and cats, improving patient quality of life, and generating insights of potential relevance to human oncology.

**Abstract:**

Drug repurposing, that is, the identification of new therapeutic indications for existing medications, has been shown to be a cost-effective and time-efficient alternative to de novo drug development. This review provides a comprehensive overview of repurposed drugs in veterinary oncology, describing their mechanisms of action, current evidence of clinical benefit, and translational relevance. The therapeutic agents discussed include non-steroidal anti-inflammatory drugs (e.g., piroxicam), metabolic modulators (e.g., metformin), anti-parasitic drugs (e.g., fenbendazole), immunomodulators (e.g., thalidomide, oclacitinib), cardiovascular agents (e.g., propranolol, statins, losartan), and other compounds such as auranofin and disulfiram. A critical evaluation of the extant evidence-based data from preclinical research, naturally occurring tumor models, and clinical studies is provided, with particular emphasis on both the therapeutic potential and the current limitations. The present review also focused on combination strategies and multimodal protocols, where repurposed drugs may enhance the efficacy of chemotherapy, targeted therapies, or immunotherapy. Challenges to clinical implementation, including limited funding, regulatory and ethical considerations, and the need for well-designed, multi-institutional clinical trials, are discussed. Ultimately, drug repurposing represents a practical and translationally valuable approach to broaden therapeutic options, improve quality of life in companion animals, and advance comparative oncology by promoting progress that benefits both veterinary and human patients.

## 1. Introduction

Drug repurposing, also referred to as drug repositioning, reprofiling, or re-tasking, is a strategic approach that explores new therapeutic indications for existing pharmacological agents, including those already approved or previously discontinued due to efficacy concerns rather than safety concerns. By leveraging compounds with well-characterized safety, pharmacokinetic, and pharmacodynamic profiles, this strategy offers significant advantages over de novo drug development, particularly in terms of reduced cost, shorter development timelines, and lower risk [1,2].

This strategy has emerged as a cornerstone in modern pharmacological innovation, offering the potential to significantly reduce development timelines and costs by leveraging existing toxicological, pharmacokinetic and pharmacodynamic data [3,4].

Between 2007 and 2009, approximately 30–40% of drugs approved by the U.S. Food and Drug Administration (FDA) were repurposed products [5]. Similarly, an analysis of transformative drugs, defined as pharmaceuticals that are both innovative and have groundbreaking effects on patient care [6], approved between 1984 and 2009 revealed that 35% were originally developed for indications distinct from their final approved use [6].

Repurposing efforts typically follow two pathways: “on-target” repurposing, where a drug’s established mechanism of action is redirected to a new indication involving the same molecular target, and “off-target” repurposing, which exploits additional, often serendipitous, pharmacologic activities. Approximately 80% of successful cases fall under the former category [4].

The rationale for drug repurposing is particularly attractive in oncology, a field characterized by high failure rates in drug development, lengthy approval processes, and an urgent need for novel therapeutic options [7,8].

Repurposed agents exhibit mechanisms that address core cancer hallmarks, including anti-inflammatory and immunomodulatory effects (e.g., piroxicam, thalidomide) [9,10], metabolic interference (e.g., metformin, disulfiram) [11,12], angiogenesis inhibition (e.g., statins, propranolol) [13,14], apoptosis induction, and cell cycle arrest (e.g., fenbendazole, mebendazole) [15].

Cancer is also a leading cause of morbidity and mortality in companion animals, particularly dogs and cats. In veterinary oncology, treatment protocols often mirror those adopted in human medicine, including surgery, chemotherapy and radiotherapy. However, the development of novel, species-specific anticancer drugs is an extremely costly and time-consuming process. Furthermore, the high cost of new-generation anticancer drugs limits accessibility for many pet owners, creating a significant unmet clinical need [1].

Within this context, drug repurposing represents a pragmatic and economically sustainable alternative, broadening therapeutic options for veterinary patients and contributing to the growing field of comparative oncology. Comparative oncology plays a pivotal role in translational cancer research. Dogs spontaneously develop tumours that closely resemble human cancers in biological behaviour, histopathology, and treatment response. Unlike laboratory models with artificially induced neoplasms, these naturally occurring tumours capture the complexity and heterogeneity of human disease, making therapeutic outcomes in dogs more predictive for human applications. This biological similarity enables a bidirectional exchange of knowledge, positioning veterinary oncology as a strategic partner in accelerating drug development for both species [16].

While many drugs may be promising candidates for repurposing, their true therapeutic potential remains uncertain. It is important for the readers to recognize that many repurposed drugs have been investigated primarily through in vitro studies (using cell cultures) or in vivo models (e.g., mice with artificially induced tumours). Although these laboratory studies serve as an essential foundation for identifying potential drug candidates, the likelihood of a drug demonstrating efficacy in vitro or in animal models translating to success in clinical trials is extremely low. In fact, only approximately 5–8% of novel anticancer drugs that exhibit effectiveness in a laboratory setting ultimately receive clinical approval for therapeutic use [17].

Careful analysis is essential when evaluating evidence presented in published studies concerning specific drugs. Greater emphasis should be placed on robust evidence of efficacy, particularly findings derived from clinical trials, or, at a minimum, clinical studies conducted on naturally occurring cancer.

Therefore, the aim of this review is to provide a comprehensive and critical overview of the current evidence regarding drug repurposing in veterinary oncology, with a focus on the pharmacological rationale, preclinical and clinical data, and translational implications for comparative oncology. By summarizing both the therapeutic opportunities and the existing limitations, this review intends to support evidence-based decision-making and highlight future research priorities in the field.

## 2. Repurposed Drugs in Veterinary Oncology

Summary of repurposed drugs investigated in veterinary oncology is shown in Table 1.

### 2.1. Non-Steroidal Anti-Inflammatory Drugs (NSAIDs): Piroxicam

Piroxicam is a non-selective cyclooxygenase COX enzyme inhibitor with more specific efficacy on COX-2 blockade. Cyclooxygenase is a growth factor found overexpressed in many cancer types, especially carcinoma in both dogs and people [18]. Piroxicam is commonly used in the treatment of canine transitional cell carcinoma (TCC), as most TCC tumours overexpress COX-2 receptors. In the larger study of dogs with TCC treated with piroxicam alone, disease stabilization was observed in 53% of cases, partial responses in 12%, and complete responses in 6% [19]. In vitro piroxicam appears to exert anticancer effects independently of its COX-2 inhibition, potentially by reducing tumor burden, promoting apoptosis, and in one study was found to reduce urinary concentrations of basic fibroblast growth factor (bFGF) [20]. Other COX-2 selective NSAIDs like deracoxib and meloxicam, have been found to be similarly effective, while other non-selective COX-2 inhibitors like carprofen are less likely to achieve a similar benefit in canine TCC treatment [21]. The established efficacy of piroxicam in canine TCC has resulted in the inclusion of COX-2 inhibitors in chemotherapy treatment regimens including both target, conventional and metronomic chemotherapy [22] (Table 1).

### 2.2. Anti-Parasitic Agents: Fenbendazole

Fenbendazole (FBZ), an inexpensive and widely available anti-parasitic drug. It has gained some interest for its potential anticancer properties. Proposed mechanisms of action include microtubule polymerization disruption, apoptosis induction, cell cycle arrest at the G2/M phase, angiogenesis inhibition, and interference with glucose and potentially glutamine metabolic pathways [15]. Some human case reports suggest potential for complete or near-complete remission in advanced cancer cases when combined with other therapies with good tolerability [23,24,25]. However, the few clinical trials that have been conducted in people with similar compounds did not demonstrate any clinical benefit [26].

Furthermore, even preclinical studies using FBZ have shown contradictory results. A murine study reported that FBZ administered as a single agent promoted tumour growth, whereas its combination with a vitamin mix led to growth inhibition [27]. This suggests that FBZ efficacy is highly context-dependent and may require specific co-treatments. The conflicting results in animal studies and reliance on anecdotal evidence highlight a critical gap in robust, controlled veterinary clinical trials for FBZ. Despite promising in vitro mechanisms, the absence of consistent in vivo evidence, together with issues of solubility and systemic exposure, suggests that uncontrolled use in companion animals may be unsafe, with risks such as hepatotoxicity and even accelerated tumour progression [15] (Table 1).

### 2.3. Anti-Diabetic Agents: Metformin

Metformin, a widely prescribed biguanide for the management of type 2 diabetes mellitus, has long attracted attention for its potential anticancer properties. In human oncology, growing preclinical and epidemiological evidence indicates that metformin may lower cancer incidence and improve clinical outcomes across several malignancies, particularly breast, colorectal, and prostate cancer [28,29,30]. The drug’s antitumor activity is thought to arise primarily through inhibition of mitochondrial complex I, resulting in energetic stress and subsequent activation of AMP-activated protein kinase, which in turn downregulates the Phosphoinositide 3-kinase/Protein Kinase B/Mammalian target of rapamycin (PI3K/AKT/mTOR) axis, a central pathway in tumour growth and metabolism [31]. Beyond its metabolic effects, metformin has been shown to exert direct antiproliferative and pro-apoptotic actions, inhibit epithelial–mesenchymal transition (EMT), reduce cancer stem cell populations, and modulate the tumour microenvironment through effects on angiogenesis and immune cell function [32,33]. Clinical trials in humans have explored its use as an adjuvant in breast cancer therapy, where metformin has been associated with improved pathological complete response rates in diabetic and, to a lesser extent, non-diabetic patients [34].

Preclinical studies in dogs have demonstrated that metformin can inhibit cell proliferation, promote apoptosis, and impair EMT in canine mammary carcinoma cell lines, with similar signalling mechanisms to those observed in human breast cancer [35,36]. In vivo, the drug reduced tumor growth in xenograft models of metastatic canine mammary carcinoma [37]. Moreover, cancer stem-like cells isolated from canine mammary tumours appear particularly sensitive to metformin, supporting its potential role in targeting chemoresistant subpopulations [38]. Clinically, the combined oral administration of cyclophosphamide and metformin has been shown to prolong median survival time in female dogs with mammary gland carcinoma, while posing a low risk of adverse effects [11]. Additional investigations have also evaluated metformin in other tumour types, including canine osteosarcoma, where it has shown activity against tumour-initiating cells and potential radiosensitizing properties [37], as well as in prostate and urothelial carcinoma cell lines [39]. In cats, in vitro experiments on injection-site sarcoma cell lines demonstrated dose-dependent cytotoxicity independent of mTOR inhibition [40], and a pilot dose-escalation clinical trial in tumour-bearing cats identified gastrointestinal toxicity and hyperlactatemia as dose-limiting, with a maximum tolerated dose of 10 mg/kg q12h [41] (Table 1).

### 2.4. Immunomodulatory Agents: Oclacitinib and Thalidomide

#### 2.4.1. Oclacitinib

Oclacitinib is an oral Janus kinase (JAK) inhibitor with predominant selectivity for JAK1, while also exerting inhibitory activity against JAK2, JAK3, and Tyrosine kinase2. Its pharmacological effect is mediated through blockade of the Janus kinase/Signal transducer and activator of transcription (JAK/STAT) signalling pathway, leading to reduced expression of several pro-inflammatory and pruritogenic cytokines, including interleukin IL-2, IL-4, IL-6, IL-13, and IL-31 [42,43]. Since the JAK/STAT axis is critical for T-cell survival and function, the immunological implications of oclacitinib have been further investigated: in vitro studies have demonstrated that the drug can induce apoptosis of canine CD4^+^ and CD8^+^ T lymphocytes and significantly suppress the production of cytokines such as IL-15 and IL-10 [44,45] suggesting a direct immunosuppressive effect that may also interfere with tumour biology. Regarding oncologic applications, current evidence is limited and largely focused on T-cell lymphomas. The efficacy of oclacitinib in cutaneous epitheliotropic lymphoma (CEL) appears limited: in a retrospective cohort of eight dogs, symptomatic improvement was documented in only one (12.5%), while the others failed to respond clinically, though adverse events remained mild [46].

Nevertheless, several case reports suggest that the drug may exert measurable—albeit transient—antitumour activity when administered at higher-than-label doses [47,48,49,50]. In a Staffordshire Bull Terrier with CEL, oclacitinib at 0.7 mg/kg BID induced a partial remission lasting approximately three months [47]. Additional reports describe durable clinical benefit in indolent T-cell subtypes, including lingual T-zone lymphoma, where long-term therapy resulted in sustained symptomatic control without conventional chemotherapy and with no notable adverse effects [48]. In a Golden Retriever with CEL, oclacitinib therapy was continued for over a year, during which the dog maintained stable clinical benefit without relevant toxicity [49]. Even more aggressive regimens have been attempted: in a case report, a dog with chemotherapy-refractory CEL tolerated escalating doses up to 3.3 mg/kg BID, achieving improved appetite, weight gain, and survival of 189 days, with only mild hematologic toxicity (anaemia and leukopenia) reported [50].

Finally, beyond lymphoma, oclacitinib has also been evaluated in tumour-bearing dogs in combination with standard chemotherapy. A pilot study demonstrated that concurrent administration with carboplatin or doxorubicin was clinically well tolerated, without evidence of unexpected synergistic toxicity, supporting the feasibility of future multimodal approaches [51] (Table 1).

#### 2.4.2. Thalidomide

Thalidomide was introduced in the 1950s as a sedative and antiemetic, but its widespread use soon became one of the most serious tragedies in the history of pharmacology, due to the severe teratogenic effects observed in human newborns [52,53,54]. Strikingly, such malformations were not reproduced in rodents, immediately raising questions regarding species specificity. The turning point came decades later, with the identification of cereblon (CRBN) as the direct molecular target of thalidomide [53,55,56]. Cereblon functions as the substrate receptor of the E3 ubiquitin ligase complex Cullin-RING LIGASE 4 Cereblon (CRL4CRBN): binding of thalidomide and its immunomodulatory derivatives (IMiDs) alters the substrate specificity of the complex, leading to degradation of transcription factors such as Ikaros Zinc Finger proteins 1 and 3 (IKZF1/3), responsible for their immunomodulatory and antineoplastic effects, but also of proteins essential for embryonic development such as spalt-like transcription factor 4 (SALL4), whose degradation accounts for teratogenicity [55,57,58]. Even subtle differences in CRBN sequence explain the marked interspecies variability: in rodents, substitution of specific residues (e.g., I391) prevents recruitment of neosubstrates, resulting in resistance to both therapeutic and teratogenic effects [56,59], whereas in humans, both pathways are active. Comparative analyses suggest that canine CRBN is more similar to the murine than to the human protein, implying that the canonical CRBN–IKZF1/3 axis may not be fully functional in dogs [55,60].

From a toxicological perspective, dogs have shown an exceptionally wide safety margin for thalidomide. Regulatory studies reported no significant systemic toxicity or tumour development even after 53 weeks of administration at extremely high doses (up to 1000 mg/kg/day; NOAEL ≈ 200 mg/kg/day) [61]. More recent pharmacokinetic investigations confirmed that clinical doses of 5–10 mg/kg once daily are generally well tolerated, with mostly mild adverse effects such as sedation, and increased risk only at cumulative doses [52,58,62]. These observations led to the hypothesis that, as in rodents, the absence of toxicity and teratogenicity in dogs might also reflect a potential lack of efficacy through the canonical CRBN-dependent pathway.

Nevertheless, experimental and clinical evidence has shown that thalidomide can still exert biological activity in dogs. Notably, a study in canine hemangiosarcoma demonstrated a reduction in Vascular Endothelial Growth Factor (VEGF) expression in metastases of thalidomide-treated dogs [63], confirming the activation of anti-angiogenic mechanisms. Interestingly, studies in humanized CRBN mice revealed that IMiDs may act through at least two distinct therapeutic pathways [56]. In these models, substitution of a single amino acid residue in murine CRBN was sufficient to restore sensitivity to IMiDs, enabling IKZF1 degradation and increased IL-2 production, thereby mimicking the canonical human pathway. At the same time, however, CRBN-independent mechanisms were also identified, suggesting that part of the therapeutic activity of thalidomide and related compounds does not rely exclusively on classical neosubstrate degradation. These alternative mechanisms include anti-angiogenic and immunomodulatory effects, such as inhibition of VEGF and FGF2 expression and suppression of Tumour Necrosis Factor-alpha signalling [60,64,65]. This evidence supports the hypothesis that, despite the likely inefficiency of the CRBN–IKZF1/3 axis in dogs, thalidomide may still exert clinically relevant activity through alternative pathways.

In human oncology, the clinical use of thalidomide in multiple myeloma has markedly declined following the introduction of second-generation immunomodulatory drugs (IMiDs), particularly lenalidomide and pomalidomide, which offer greater efficacy and a more favourable hematologic toxicity profile [66,67]. Lenalidomide has become the standard of care for maintenance therapy after autologous stem cell transplantation (ASCT) in standard-risk patients, while combination strategies with bortezomib are recommended for those with high-risk cytogenetics [66,67].

Thalidomide now has a restricted role, mainly in settings where lenalidomide is unavailable, contraindicated, or poorly tolerated. Its continued value is partly related to its lack of requirement for dose adjustment in patients with renal impairment and its ability to promote renal recovery in a subset of cases [68]. Nevertheless, its long-term use is limited by cumulative toxicities—most notably peripheral neuropathy—and by inferior outcomes compared with lenalidomide-based regimens [67].

In veterinary oncology, the rationale for thalidomide use derives mainly from its anti-angiogenic and anti-inflammatory properties. In a prospective study including 15 dogs with splenic hemangiosarcoma, thalidomide administration after splenectomy resulted in a significant prolongation of median survival compared with surgery alone [69] with histological evidence of reduced VEGF expression in metastases [63]. In canine mammary carcinomas, multimodal protocols including thalidomide and/or metronomic chemotherapy have shown clinical benefits in terms of disease control and survival [10]. Thalidomide has also been evaluated in combination protocols for canine pulmonary carcinoma, where metronomic cyclophosphamide, piroxicam, and thalidomide were associated with good tolerability and promising survival outcomes [1,70]. More recently, a multi-institutional retrospective study investigated thalidomide as a rescue therapy in dogs with multiple myeloma (MM) refractory to alkylating agents. The treatment was well tolerated and achieved durable clinical responses, supporting its potential role as a rescue approach in canine MM [71] (Table 1).

### 2.5. Cardiovascular Drugs: Statins, Propranolol and Losartan

#### 2.5.1. Statins

Statins, inhibitors of 3-hydroxy-3-methylglutaryl-coenzyme A reductase, are primarily cholesterol-lowering agents, but exhibit pleiotropic effects, including antiproliferative, pro-apoptotic, and anti-invasive properties in tumour cells in vitro [13]. They can show synergistic effects with chemotherapeutic agents and radiotherapy, even in chemoresistant tumours. The underlying mechanism appears to involve the inhibition of the mevalonate pathway, which normally generates small lipid intermediates known as isoprenoids (farnesyl and geranylgeranyl pyrophosphate). These molecules are required for protein prenylation, a post-translational modification that enables small Guanosine-5′-triphosphate-binding proteins such as RAS, RHO, and RAC to attach to the plasma membrane and activate intracellular signalling cascades regulating proliferation, migration, and survival. By reducing isoprenoid synthesis, statins prevent protein prenylation, thereby disrupting oncogenic signalling and promoting apoptosis [72,73].

In human breast cancer models, statin exposure has been shown to reduce the expression of CD44, a surface adhesion molecule critical for the maintenance and drug-resistant phenotype of cancer stem cells. This modulation has been associated with decreased invasiveness, loss of stem-like features, and enhanced chemosensitivity [74,75,76].

In veterinary oncology, similar findings have been observed: simvastatin, a lipophilic statin, demonstrated in vitro antiproliferative effects on canine mammary carcinoma stem-like cells and increased their sensitivity to doxorubicin, supporting its potential as a novel therapeutic option for this tumor type [77].

This suggests that statins could address a major challenge in oncology, drug resistance and recurrence, by eliminating the subpopulation of cells responsible for tumour initiation and maintenance. This positions statins not merely as generic antiproliferative agents but as potential precision medicine tools when combined with conventional therapies (Table 1).

#### 2.5.2. Propranolol

Propranolol, a non-selective β-adrenergic receptor antagonist, exhibits antitumor effects, particularly against tumours originating from vascular endothelial cells. In vitro studies have shown that propranolol sensitizes vascular sarcoma cells to doxorubicin by altering lysosomal drug sequestration and efflux, thereby increasing intracellular anthracycline accumulation [14].

The antitumour activity of propranolol appears to be multifactorial. Beyond its vasoconstrictive and antiangiogenic properties, propranolol inhibits catecholamine-mediated activation of β-adrenergic receptors, which are known to promote tumour cell proliferation, migration, and angiogenesis through the cyclic adenosine monophosphate–protein kinase A (cAMP–PKA) and mitogen-activated protein kinase/extracellular signal–regulated kinase (MAPK/ERK) signalling pathways [78,79]. Inhibition of β-adrenergic signalling also modulates the tumour microenvironment, reducing stress-induced release of proangiogenic and immunosuppressive mediators [80]. Moreover, propranolol may enhance chemotherapy efficacy by impairing lysosomal drug sequestration, decreasing P-glycoprotein–mediated efflux, and potentially contributing to tumour vascular normalization, which improves drug delivery and reduces hypoxia-driven resistance [14,80]. This combined modulation of tumour microenvironment and intracellular pharmacokinetics likely contributes to the observed synergy with anthracyclines.

Clinically, promising results have been reported in canine hemangiosarcoma. In a pilot study involving five dogs with stage III hemangiosarcoma, the combination of propranolol and anthracyclines achieved clinical benefit in 80% of cases, including partial regression of metastatic lesions, with an adverse-event profile comparable to anthracycline monotherapy and no serious or irreversible toxicities [81]. More recently, a multi-institutional retrospective study of twelve dogs with advanced or metastatic hemangiosarcoma receiving propranolol (0.5–1.1 mg/kg PO q8–12h) combined with dose-intense chemotherapy reported disease control in 83% of cases (10/12), a median time-to-progression of 66 days, and a median overall survival of 83 days, with no grade ≥ 3 adverse events [82] (Table 1).

#### 2.5.3. Losartan

Losartan, an angiotensin II receptor blocker commonly prescribed for hypertension and renal disease, has emerged as a promising candidate for repurposing in oncology due to its ability to modulate the tumour microenvironment. Angiotensin II promotes stromal fibrosis, angiogenesis, and immunosuppression, all of which contribute to tumour progression. Preclinical work demonstrated that losartan inhibits collagen I synthesis, reduces stromal density, and thereby improves drug penetration and oxygen delivery in solid tumours [83]. Beyond its antifibrotic (stromal modulating) effects, inhibition of the angiotensin II/AT1R axis has been associated with reduced inflammatory signalling, lower macrophage infiltration, and attenuation of immunosuppressive pathways within the tumour microenvironment [84,85].

Overall, the available evidence suggests that losartan exerts indirect immunomodulatory effects that act synergistically with its stromal-normalizing properties, potentially enhancing therapeutic outcomes and tumour perfusion [86]. In veterinary oncology, losartan has been evaluated in dogs with naturally occurring tumours. A multicentre study in metastatic appendicular osteosarcoma showed that high-dose losartan combined with toceranib achieved an objective response rate of 25% (4/16 dogs with partial response), and an overall clinical benefit rate of approximately 50% when including dogs with durable stable disease (>8 weeks) [86]. More recently, a prospective open-label pilot trial in canine gliomas combined oral losartan and propranolol with a cancer stem cell vaccine, achieving an 80% clinical benefit rate (partial response in 2 dogs, stable disease in 6) and a median overall survival of 351 days. Importantly, dogs that developed anti-CSC antibody responses had significantly longer survival, supporting the immunomodulatory impact of this multimodal approach [87] (Table 1).

### 2.6. Other Repurposed Agents (e.g., Auranofin, Desmopressin, Disulfiram)

#### 2.6.1. Auranofin

Auranofin is a drug originally approved for the treatment of rheumatoid arthritis in humans, which has recently demonstrated anticancer potential in vitro. Its primary mechanism of action involves the inhibition of thioredoxin reductase, resulting in the intracellular accumulation of reactive oxygen species (ROS) and induction of tumour cell death. Preclinical studies have shown that auranofin impairs proliferation, invasiveness, and metastatic dissemination of osteosarcoma cells in vitro and in murine models [88].

A single-arm, multicentre phase I/II clinical trial in dogs with appendicular osteosarcoma evaluated the addition of auranofin to standard therapy consisting of limb amputation and carboplatin chemotherapy. The combination conferred a survival benefit compared to historical controls, with the effect being particularly marked in male dogs. Notably, 25% of treated dogs were still alive between 806 and 1525 days after treatment, suggesting a durable clinical response [81]. These findings support the potential role of auranofin as an adjunctive agent in canine osteosarcoma and highlight its value within a comparative oncology framework (Table 1).

#### 2.6.2. Desmopressin

Desmopressin (DDAVP) is a synthetic derivative of an antidiuretic hormone (vasopressin), used to treat diabetes insipidus and coagulation disorders. Its proposed anti-metastatic effect appears related to its ability to inhibit metastatic emboli formation and their adhesion to target metastatic sites. However, studies in canine mammary carcinoma have yielded conflicting results.

One prospective randomized trial involving 24 dogs found no significant difference in disease-free interval (DFI) or median survival time (MST) between treated and control groups [89]. In contrast, another study in dogs with high-grade mammary tumours reported significantly prolonged DFI and MST following intravenous administration of DDAVP [90]. In vitro research on the canine mammary carcinoma cell line also showed modest antiproliferative effects at high concentrations [91]. Conversely, a retrospective study in cats with mammary carcinoma found no survival benefit from DDAVP administration [92] (Table 1).

#### 2.6.3. Disulfiram

Disulfiram (DSF), an aldehyde dehydrogenase inhibitor approved for the treatment of alcohol dependence, has gained considerable attention as a repurposed anticancer agent. After conversion to diethyldithiocarbamate (DTC), DSF displays context-dependent redox activity: in reducing/low-copper settings, DTC can scavenge reactive species and mitigate oxidative injury to normal tissues, whereas in copper-replete tumor microenvironments, DSF rapidly forms copper complexes (e.g., copper diethyldithiocarbamate complex, CuET) that promote ROS generation and proteotoxic stress in cancer cells. This duality reconciles reports of antioxidant protection in normal tissues with pro-oxidant cytotoxicity and radiosensitization in tumours [93,94]. Its antitumor activity has been documented across in vitro, animal, and human studies, with early clinical observations suggesting potential benefit [95].

Beyond redox effects, CuET targets protein homeostasis by aggregating the p97/valosin-containing protein (VCP) adaptor nuclear protein localization protein 4 NPL4, blocking the ubiquitin–proteasome system and amplifying ER-stress–mediated apoptosis [96]. Preclinical studies show tumour growth inhibition and survival gains, enhanced when combined with copper or delivered via nanoparticles [96,97,98,99]. Notably, DSF can also act as a radiosensitizer in the absence of exogenous copper, increasing ionizing radiation-induced DNA double-strand breaks, G2/M arrest, and apoptosis, while sparing normal tissues [93,94]. An immunomodulatory role has been proposed as well, as DSF/Cu can induce immunogenic cell death and potentiate immune checkpoint blockade in murine models [97,98]. In veterinary oncology, evidence remains limited. To date, a study in canine mammary tumours reported that DSF reduced proliferation/migration and induced apoptosis via PI3K/Akt/mTOR suppression (IC_50_ ≈ 97 nM), but Mucin 1 (MUC1) overexpression attenuated these effects in vitro and in xenografts, underscoring the impact of tumour molecular context on DSF responsiveness in companion animals [100] (Table 1).

**Table 1:** Summary of repurposed drugs investigated in veterinary oncology. Abbreviations: ALDH, aldehyde dehydrogenase; AMPK, adenosine monophosphate–activated protein kinase; AT1R, angiotensin II type 1 receptor; COX, cyclooxygenase; CRBN, cereblon; CSC, cancer stem cell; CR: complete response; DFI, disease-free interval; EMT, epithelial–mesenchymal transition; FGF2, fibroblast growth factor 2; GI, gastrointestinal; HMG-CoA, 3-hydroxy-3-methylglutaryl-coenzyme A; IL, interleukin; JAK, Janus kinase; MAPK/ERK, mitogen-activated protein kinase/extracellular signal-regulated kinase; MM, multiple myeloma; mTOR, mammalian target of rapamycin; MST, median survival time; NPL4, nuclear protein localization protein 4; OS, overall survival; PFS, progression-free survival; PI3K/AKT, phosphoinositide 3-kinase/protein kinase B; PR: partial response; ROS, reactive oxygen species; SD: stable disease; STAT, signal transducer and activator of transcription; TCC, transitional cell carcinoma; TTP, time-to-progression; VEGF, vascular endothelial growth factor.

Repurposed Drug	Action/Mechanism	Clinical Trial Study Type	Clinical or Experimental Results	Statistics Outcomes	References
Piroxicam	Non-selective COX inhibitor; reduces COX-2–mediated prostaglandin synthesis; promotes apoptosis and anti-angiogenic effects.	Clinical studies in dogs with TCC.	53% SD, 12% PR, 6% CR; often used in combination or metronomic chemotherapy.	MST ≈ 181–244 days depending on protocol.	[19,20,21,22]
Fenbendazole	Microtubule polymerization inhibitor; induces G2/M arrest, apoptosis, anti-angiogenic and metabolic interference (glucose/glutamine).	Preclinical and anecdotal reports; no controlled veterinary clinical trials.	Contradictory results: tumour inhibition in some models, tumour promotion in others; potential hepatotoxicity.	Not applicable.	[15,25,26]
Metformin	Inhibits mitochondrial complex I → AMPK activation → PI3K/AKT/mTOR suppression; anti-proliferative, anti-EMT, anti-stemness.	In vitro canine mammary carcinoma; xenografts; clinical pilot studies.	Decreased proliferation, increased apoptosis, and suppressed EMT; extended MST with cyclophosphamide; radiosensitization observed in osteosarcoma models.	MST prolongation in treated dogs; dose-limiting GI toxicity and hyperlactatemia in cats.	[11,36,38,41]
Oclacitinib	JAK1 > JAK2/3 inhibitor; suppresses IL-2, IL-4, IL-6, IL-13, IL-31; immunomodulatory and potential antineoplastic effects via T-cell suppression.	Retrospective and case reports in dogs with cutaneous epitheliotropic lymphoma and T-zone lymphoma.	PR and long-term stabilization in few cases; mild hematologic toxicity.	Median duration of response is 3–6 months.	[47,48,49,50,51]
Thalidomide	Anti-angiogenic, anti-inflammatory; inhibits VEGF and FGF2; possible CRBN-independent immunomodulation.	Prospective trial in dogs with splenic hemangiosarcoma; multimodal studies in mammary and pulmonary carcinoma. Retrospective study in canine MM	Prolonged MST vs. surgery alone; reduced VEGF in metastases; good tolerability. Durable responses in MM.	MST 184 vs. 133 days (*p* < 0.05); median PFS 298 days; OS 630 days.	[10,52,63,69,71]
Statins (e.g., Simvastatin)	HMG-CoA reductase inhibition → reduced isoprenoid synthesis → impaired protein prenylation (RAS, RHO, RAC); antiproliferative, anti-invasive.	In vitro studies in canine mammary carcinoma stem-like cells.	Reduced proliferation and CD44 expression, with enhanced sensitivity to doxorubicin.	Not applicable.	[74,75,77]
Propranolol	β-adrenergic receptor blockade; reduced cAMP/PKA and MAPK/ERK signalling; anti-angiogenic, chemosensitizing.	Pilot and retrospective studies in dogs with hemangiosarcoma.	Clinical benefit in 80%; partial regression in metastatic cases; well tolerated with anthracyclines.	MST ≈ 83 days; TTP ≈ 66 days.	[80,81,82]
Losartan	AT1R blockade → antifibrotic, anti-inflammatory, immunomodulatory; normalizes tumour stroma and perfusion.	Multicentre osteosarcoma and glioma studies in dogs.	PR in 25%; clinical benefit rate 50%; synergistic effect with toceranib, propranolol, and CSC vaccine.	Median OS 351 days in glioma trial.	[85,86,87]
Auranofin	Inhibits thioredoxin reductase, leading to increased ROS and oxidative stress and induction of apoptosis.	Phase I/II clinical trial in dogs with osteosarcoma.	MST 329 vs. 240 days (control); benefit particularly in males.	*p* = 0.036.	[81,88]
Desmopressin	Anti-metastatic; inhibits emboli formation and adhesion to secondary sites; modulates V2 receptors.	Randomized clinical trials in dogs with mammary carcinoma.	Prolonged DFI and OS in some studies; variable reproducibility.	DFI 608 vs. 85 days; OS > 600 vs. 333 days (*p* < 0.01).	[89,90,92]
Disulfiram	ALDH inhibitor; Cu-dependent ROS generation; p97/NPL4 aggregation → proteotoxic stress, apoptosis; radiosensitizer.	In vitro and xenograft studies on canine mammary carcinoma.	Reduced proliferation and migration; apoptosis induction via PI3K/Akt/mTOR inhibition; resistance associated with MUC1 overexpression.	IC_50_ ≈ 97 nM.	[93,94,100,101]

## 3. Synergistic Approaches and Combination Therapies

Combination therapies are crucial in oncology, as a single drug may not be effective against heterogeneous tumours. Combining drugs increases the likelihood and durability of response by targeting different subsets of tumour cells. Repurposed drugs can act as chemosensitizers, increasing the intracellular accumulation and efficacy of conventional chemotherapeutic agents. Examples include piroxicam in metronomic chemotherapy, metformin with cyclophosphamide [13], auranofin with carboplatin [1], propranolol with anthracyclines, and statins sensitizing cells to doxorubicin. Repurposed drugs can also synergize with immunotherapies, for instance, by reducing platelet activity (aspirin) or counteracting immunosuppressive cells in the tumour microenvironment (combination of propranolol, losartan, toceranib) [1]. The recurring theme of synergy between repurposed drugs and emerging therapeutic strategies suggests that the future of veterinary oncology lies not in single-agent solutions, but in rationally designed multimodal combination therapies. This approach aims to exploit complementary mechanisms—such as anti-angiogenesis, immunomodulation, and direct cytotoxicity—to overcome tumour resistance and achieve more durable clinical outcomes, in alignment with trends observed in human oncology.

## 4. Challenges and Future Directions

### 4.1. Challenges in Clinical Trials for Drug Repurposing

Clinical trials in veterinary oncology, particularly those investigating repurposed drugs, often encounter significant funding limitations compared to human oncology, which can hinder the design and execution of large-scale, well-controlled studies. Recruiting enough eligible patients is also challenging due to the inherent heterogeneity of spontaneous tumours in companion animals, variability in owner consent and compliance, and the wide geographical dispersion of potential candidates. The high clinical heterogeneity of enrolled patients with respect to tumour stage represents a major limitation [102]. Many veterinary studies, especially those on repurposed agents, are observational or case series, often lacking placebo or standard-of-care-alone control arms, which reduces the robustness of the evidence. Moreover, the absence of standardized treatment protocols for some off-label therapies results in inconsistent and unpredictable clinical responses.

Another important consideration is that traditional outcome measures borrowed from human oncology, such as MST, are strongly influenced by the decision to perform euthanasia, which may not directly reflect treatment efficacy. For this reason, surrogate endpoints such as progression-free survival (PFS), time to progression (TTP), or overall response rate (ORR) are often more reliable in veterinary trials. In addition, quality of life (QoL) represents a critical outcome parameter, frequently prioritized over the mere extension of survival. This reflects a paradigm shift also occurring in human oncology, where increasing attention is being paid to QoL in drug approval processes. In veterinary medicine, a treatment that prolongs life for only a short period while significantly worsening QoL would generally be considered unacceptable for dogs or cats, underscoring the ethical emphasis on preserving well-being alongside survival time.

From both a translational and economic standpoint, the expiration of patents and the limited market value of many repurposed drugs substantially reduce commercial incentives to support rigorous clinical trials for new oncologic indications [103].

The confluence of limited funding, patient heterogeneity, and lack of commercial incentives creates a significant bottleneck for the advancement of repurposed drugs in veterinary oncology through rigorous clinical trials [1]. Addressing this challenge requires innovative research strategies, including multi-institutional collaborations, adaptive trial designs, and expanded support through public and private funding mechanisms, to generate the high-quality evidence necessary for widespread clinical adoption.

### 4.2. Regulatory and Ethical Considerations for Off-Label Drug Use

The off-label use of authorized veterinary medicinal products (i.e., their use outside the approved conditions, such as for a different indication, species, or dosage) is a common and generally legal practice in both human and veterinary medicine [104]. When supported by scientific evidence or expert clinical judgment, it is often considered part of the standard of care.

In the European Union, Regulation (EU) 2019/6, which came into force in January 2022, represents the most up-to-date and comprehensive regulatory framework on veterinary medicinal products. Among its key provisions, it imposes stricter limitations on off-label prescribing, particularly concerning dosage alterations and indication deviations, to enhance responsible use and harmonized pharmacovigilance across member states [105].

In the United States, the Animal Medicinal Drug Use Clarification Act (AMDUCA) allows off-label use under specific conditions, including the presence of a valid veterinarian-client-patient relationship and proper documentation of the treatment rationale and outcomes [106].

Veterinarians have an ethical obligation to act in the best interests of their patients and clients. When prescribing a drug off label, they must ensure the owner is adequately informed about the animal’s clinical condition, available treatment options, associated risks, prognosis, and estimated costs. Written informed consent is strongly recommended, especially when the treatment is experimental or carries a degree of uncertainty.

While off-label use can offer therapeutic flexibility, it also carries potential risks—including treatment failure, unexpected adverse effects, and the absence of formal safety or efficacy evaluation for the specific use. This places the responsibility for risk–benefit assessment directly on the prescribing veterinarian. Continuous pharmacovigilance is therefore essential to monitor and report any adverse drug reactions arising from such practices.

In fields like veterinary oncology, where approved therapeutic options are often limited, off-label prescribing is widespread and frequently necessary. However, this creates a complex ethical and legal environment, emphasizing the need for transparency, robust informed consent processes, and accessible, peer-reviewed scientific evidence to support clinical decision-making and protect both animal welfare and professional accountability.

## 5. Conclusions and Future Perspective

Drug repurposing offers a viable and increasingly explored strategy in veterinary oncology, providing cost-effective and accessible treatment options by leveraging existing knowledge of drug safety and pharmacokinetics. A range of repurposed drugs, from NSAIDs to cardiovascular agents, demonstrate antitumor potential, often through different mechanisms targeting tumour growth, angiogenesis, and immune modulation, particularly when integrated into multimodal treatment protocols.

Despite these advancements, significant knowledge gaps persist. Large-scale, prospective, randomized clinical trials specifically in both human and veterinary medicine are lacking for many repurposed drugs, leading to variable evidence. There is an insufficient understanding of optimal dosages and long-term safety profiles for repurposed drugs when used for oncologic indications in non-primary species or indications. Research into the precise synergistic mechanisms of combination therapies involving repurposed drugs and novel modalities is limited. Standardization of protocols and reporting guidelines for veterinary clinical trials is needed to improve data quality and comparability.

Moving forward, it is crucial to prioritize well-designed, multi-institutional veterinary clinical trials to generate high-level evidence for repurposed drugs and emerging therapies. For example, large prospective clinical trials focusing on combination strategies that leverage synergistic mechanisms, integrating repurposed agents with conventional treatments, and immunotherapies will play a pivotal role. Finally, fostering continuous collaboration between veterinary and human oncology researchers through comparative oncology initiatives is imperative to accelerate translational discoveries and improve outcomes for both animal and human patients.

## Data Availability

No new data were created or analyzed in this study. Data sharing is not applicable to this article.
